# Leptospirosis seropositivity and its serovars among cattle in Northeastern Malaysia

**DOI:** 10.14202/vetworld.2018.840-844

**Published:** 2018-06-23

**Authors:** Aziah Daud, Nik Mohd Hafiz Mohd Fuzi, Mohd Mokhtar Arshad, Suratan Kamarudin, Wan Mohd Zahiruddin Wan Mohammad, Fairuz Amran, Nabilah Ismail

**Affiliations:** 1 Department of Community Medicine, School of Medical Sciences, Universiti Sains Malaysia Health Campus, Kota Bharu, Kelantan, Malaysia; 2 Faculty of Veterinary Medicine, Universiti Malaysia Kelantan, Taman Bendahara, Kota Bharu, Kelantan, Malaysia; 3 Department of Veterinary Services Kelantan, Kota Bharu, Kelantan, Malaysia; 4 Infectious Disease Research Centre, Institute for Medical Research, Kuala Lumpur, Malaysia; 5 Department of Microbiology, School of Medical Sciences, Universiti Sains Malaysia Health Campus, Kota Bharu, Kelantan, Malaysia

**Keywords:** cattle, leptospirosis, microscopic agglutination test, serovar Sarawak

## Abstract

**Background:**

Leptospirosis is a zoonotic disease that infects human and livestock which causes economic losses to the farmers. It has been reported as one of the causes of reproductive failure in cattle and other ruminants, determining abortions, stillbirth, weak newborns, and decrease in their growth rate and milk production.

**Aim:**

The objectives of this study were to determine the leptospirosis seroprevalence and to identify the predominant infecting serovars among cattle.

**Materials and Methods:**

A cross-sectional study involving 420 cattle from six randomly selected districts in Kelantan was conducted. A serological test using the microscopic agglutination test was conducted in the Institute of Medical Research with a cutoff titer for seropositivity of ≥1:100.

**Results:**

The overall prevalence of leptospirosis seropositivity among cattle in this study was 81.7% (95% confidence interval: 63.5, 80.1). The most common reaction obtained with the sera tested was from the serovar Sarawak with 78.8%.

**Conclusion:**

A high seroprevalence of leptospiral antibodies was found among cattle in Northeastern Malaysia. These findings urge that more studies are required to determine the reasons for the high seroprevalence among the cattle along with its transmission and pathogenicity of the local serovar Sarawak.

## Introduction

Leptospirosis affects humans and a variety of animal species, giving rise to significant health problems and potential fatalities. In cattle and other ruminants, leptospirosis has been described as one of the reasons for failure of reproduction, abortions, miscarriages, weak progenies, reduced weight gain, and decrease in growth rate as well as milk production [1]. Certain vertebrate animals, especially mammalian, such as cattle, buffaloes, horses, sheep, goat, pigs, dogs, and rodents are natural hosts for pathogenic leptospires that house themselves in the kidneys of the hosts. These leptospires do little or no detectable harm in the host bodies and merely maintain the infection in their hosts. Hence, animals infected by leptospires are known as natural maintenance hosts [2].

Several studies also reported a significant association between human leptospirosis and livestock [3,4]. According to the previous studies, high leptospirosis seropositivity rates were found in domestic animals (i.e., buffaloes, cattle, and pigs) which were maintenance hosts for *Leptospira* serovar Hardjo and Pomona [5,6]. In spite of the efforts to overcome leptospirosis, the disease is still prevalent in both humans and animals. It is also considered as one of the important public health re-emerging zoonosis. Hence, the knowledge of the distribution of *Leptospira* serovars and their maintenance host gives an important significance to understand the epidemiology of the disease and how it can be spread to other animals and also human.

There is still lack of data on the leptospirosis, seroprevalence and its predominant serovars among cattle in Northeastern Malaysia. Therefore, the aim of this study was to determine the leptospirosis, seroprevalence and to identify the predominant infecting serovars among cattle in Northeastern Malaysia.

## Materials and Methods

### Ethical approval

All clinical samples in this study were collected as per standard sample collection procedure without giving any stress or harm to the cattle. This study was approved ([USM/Animal Ethics Approval/2015/(97)(690)]) by the Animal Ethics Committee Universiti Sains Malaysia (USM) (AECUSM), USM.

### Informed consents

Blood sample collections from cattle were done by trained staff. Selected cattle farmers were approached and talk session was held to explain details of the research before the study was conducted. We had explained the informed consent individually to the cattle farmer before informed consents were taken.

### Study design and population

A cross-sectional study was conducted involving six districts in Kelantan which is the Northeastern state of Malaysia. Simple random sampling was applied to select six districts from the total of 10. The list of all cattle farms available was acquired from the Department of Veterinary Services. Then, stratified random sampling was applied to the list to determine the number of cattle needed to be selected from each district.

Selected farms and the cattle farmers were approached and explained regarding our study. The explanation was given to all the cattle owners about the procedure. Reference populations of this study are the cattle, where the source population is taken from multistage random sampling of cattle farms from 10 districts in Kelantan. The sampling frame is the list of cattle farms from the six districts in Kelantan who fulfill the study criteria.

Calculating based on a 36.0% seroprevalence of leptospirosis among dairy cattle [7] and 30% non-response rate, the estimated sample size required for the study was 460. The samples were taken from 120 herds with four cattle randomly selected from 100 herds and three cattle from another 20 herds. Inclusion criteria were cattle farms which need to have a travis for cattle’s blood taking procedure. Cattle whose owners not registered to the Department of Veterinary Service were excluded from the study.

### Blood samples and serological tests

About 10 mL of blood was collected from each cattle for the study. The cattle’s venous blood samples were tested for the presence of anti-leptospiral antibodies using microscopic agglutination test (MAT) at the Institute of Medical Research (IMR) following standard methods [8]. The MAT was done with a panel of live leptospire reference cultures obtained from the Royal Tropical Institute (World Health Organization/Food and Agriculture Organization of the United Nations Collaborating Centre for Reference and Research on Leptospirosis) in Amsterdam (Australis, Autumnalis, Batavia, Canicola, Celledoni, Grippotyposa, Icterohemorrhagiae, Javanica, Pomona, Pyrogenes, Hardjoprajitno, Patoc, Tarassovi, and Djasiman) and from the IMR (Melaka, Terengganu, Sarawak, Lai, Hardjo bovis, and Copenhagen).

Live leptospire cell suspensions with 20 serovars each were added to serially diluted serum specimens in wells of microtiter plates and were incubated at 30°C for 2 h. Using the control well for comparison, agglutination was examined by observing free leptospires in each well using dark field microscopy. The MAT results were considered positive if the free leptospire approximate numbers were <50% in the control well. A titer of ≥1:100 was used as the cutoff titer for leptospirosis seropositive as the level of titer indicated previous exposure to the *Leptospira* bacteria [9].

### Statistical analysis

Data were entered and analyzed using IBM Statistical Program for the Social Sciences (SPSS) version 22 for Windows [10]. All continuous variables were presented using means and standard deviations. Frequencies and percentages were used to describe categorical variables. Seroprevalence of leptospirosis was described with 95% confidence interval (CI).

## Results

We were only able to collect 420 of 460 blood samples from cattle yielding a response rate of 91.3%. The overall prevalence of leptospirosis seropositivity among cattle in this study was 81.7% (95% CI: 63.5, 80.1). Table-1 shows the prevalence of leptospirosis seropositivity among cattle by district. The district with the highest prevalence was District A where all the cattle were found to be seropositive. Other districts also had a high seropositivity prevalence which was between 74.7% and 87.5%.

**Table-1:** Seroprevalence of leptospirosis seropositivity among cattle according to district (n=420).

District	n	MAT≥1:100	95% CI

		Frequency (%)	
District A	39	39 (100.0)	88.8, 100.0
District B	72	63 (87.5)	77.1, 93.8
District C	111	92 (82.9)	74.3, 89.1
District D	83	63 (78.8)	65.0, 84.3
District E	40	30 (75.0)	58.5, 86.8
District F	75	56 (74.7)	63.1, 83.7

MAT=Microscopic agglutination test, CI=Confidence interval

The distribution of serovars among 343 seropositive cases determined by the positive MAT titer ≥1:100 is portrayed in Table-2. The most common reaction obtained with the sera tested was the *Leptospira* strain isolated from the serovar Sarawak with 78.8%, followed by serovar Patoc with 9.0 %. All other serovars were all below 2.5%.

**Table-2:** Serovar distribution among 343 seropositive cattle determined by positive MAT (titer≥1:100).

Serovars tested	Frequency (%)
Sarawak	331 (78.8)
Patoc	38 (9.0)
Hardjobovis	10 (2.4)
Australis	10 (2.4)
Hardjoprajitno	7 (1.7)
Melaka	6 (1.4)
Terengganu	6 (1.4)
Tarrasovi	6 (1.4)
Pomona	5 (1.2)
Copenhageni	4 (1.0)
Grippotyphosa	3 (0.7)
Autumnalis	2 (0.5)
Lai	1 (0.2)
Batavia	1 (0.2)
Pyrogenes	1 (0.2)
Canicola	1 (0.2)
Djasiman	1 (0.2)
Icterohemorrhage	1 (0.2)

*Cattle tested may be positive to one or more serovars. MAT=Microscopic agglutination test

The majority (63.8%) of the cattle which was tested positive for MAT result showed a reaction to only one serovar, and the rest reacted multiple serovars. The highest numbers of serovars detected were six, but it was only in one cow. Those serovars were Sarawak, Patoc, Hardjoprajitno, Tarrasovi, Melaka, and Terengganu. Second highest multiple serovars were 4 serovars detected in 3 cattle (0.7%), followed by 3 serovars in 6 cattle (1.4%), and finally 2 serovars in 65 cattle (15.5%).

## Discussion

The prevalence of leptospirosis seropositivity among cattle in this study, which was also determined by MAT titers of ≥1:100, was very high. The most common isolated serovar was Sarawak. In fact, the seropositive prevalence rate was higher compared to previous researches. A cross-sectional serological study on domestic animals in West Malaysia showed a seropositive prevalence rate of 40.5% among cattle, 31% in buffaloes, and 16% in pigs [6]. Meanwhile, another serological survey in four dairy cattle farms in Malaysia also discovered that 36% of the dairy cattle examined had leptospiral infection [7]. A leptospirosis seropositivity prevalence of 30% among cattle was also reported in another study conducted at cattle farms in Malaysia [11]. A more recent leptospirosis seropositive prevalence study conducted at adopted farms and the agricultural farm of Universiti Putra Malaysia (UPM) revealed a prevalence of 20% in the cattle [12].

All those studies used MAT to determine the leptospirosis seropositivity prevalence in the cattle. The highest number of serovars tested among those studies was 16, which were Australis, Autumnalis, Ballum, Bataviae, Canicola, Celledoni, Djasiman, Hardjobovis, Hardjoprajitno, Hebdomadis, Hurstbridge, Icterohemorrhagiae, Javanica, Pomona, Pyrogenes, and Sejroe [12]. However, it should be highlighted here that serovar Sarawak, which is a local serovar antigen, was not used as one of the test antigens in MAT in all those studies. This is most probably the main reason why the seropositive prevalence rate in our study was much higher compared to the other aforementioned studies. This is also one of the most interesting findings in our research as the presence of serovar Sarawak in cattle has not been reported before.

Outside of Malaysia, various serovars have been found in cattle, depending on the geographical location. For example, Thailand has recorded a seropositive prevalence of 9.9% in cattle, with the most commonly detected antibodies being those for *Leptospira interrogans* serovar Ranarum (31.8%) and serovar Sejroe (31.1%) [13]. The predominant serovars in cattle were *Leptospira borgpetersenii* serovar Hardjo (81.25%) in Chile [14], serovars Bratislava (7.9%) and Grippotyphosa (7.7%) in Spain [15], serovar Canicola (11.7%) in Iran [16], and serovars Hardjo and Castellonis (26.1% each) in Brazil [17].

Due to past exposure, cattle also can be seropositive for one or more serovars. In our study, more than three-quarters of seropositive cattle only had antibodies to one serovar. There were 18 serovars identified in this study, with the most common serovar Sarawak, followed by Patoc and Hardjobovis. The least common ones were Lai, Batavia, Pyrogenes, Canicola, Djasiman, and Icterohemorrhge. This differed from previous findings by other researchers. Bejo [11] reported that the serological prevalence of *Leptospira* serovar Hardjo in cattle was 30%. Under experimental condition, she also demonstrated that cattle are able to maintain serovar Hardjo. It is evident that serovar hardjo infection is present in cattle farms in Malaysia [11].

Another serological survey in Bangladesh was conducted to determine the seroprevalence and risk factors of leptospirosis in commercial dairy cattle from April 2011 to September 2012. The study was carried out by randomly selected six farms having 206 dairy cows. A total of 110 serum samples were collected for the detection of *Leptospira* serovar Hardjo antibody by ELISA. The results showed a seroprevalence of 47.27% [18]. While in India, other researcher reported the overall seroprevalence in a bovine population of 41.0%. A total of 575 serum samples (171 cattle, 245 buffaloes, 81 bullocks, and 78 bulls) randomly collected and tested with MAT (titer of 1:100) using live antigens of 18 references *Leptospira* serovars. The predominant *Leptospira* antibodies were Australis (23.61%) followed by Hardjo (19.44%) [19]. However, many of researcher did not test for serovar Sarawak and its occurrence among cattle has never been reported.

Several studies have been conducted in other countries to identify the commonly occurring serovars in humans and animals. One of them was a leptospirosis seropositivity study conducted in Thailand on humans and livestock (buffaloes, cattle, and pigs). Blood samples were collected between January 2010 and December 2015 under a passive surveillance program. Using MAT (with cutoff titer of ≥1:100), it was found that the seropositive prevalence was 23.7% in humans, 24.8% in buffaloes, 28.1% in cattle, and 11.3% in pigs. The most predominant serovars in humans were Shermani, followed by Bratislava and Panama. In buffaloes, serovars Shermani, Ranarum, and Tarassovi were the most common. While in cattle and pigs, the most common serovars were serovars Shermani and Ranarum [20].

However, another study conducted at 27 dairy farms in Brazil found a different set of predominant serovars in human and animals. The samples taken were blood and kidney from rodents, blood and urine from bovines, and blood from workers. MAT was done for serology, and it was reported that the leptospirosis seropositivity prevalence in humans was 23.53%, while that in bovines was 32.85%. In human samples, serovar Bratislava occurred most frequently (37.51%), while in bovine samples, the most frequent serovars were Hardjo and Castellonis (26.08% each). Contrary to expectations, all the rodents had negative serology results and kidney samples were also negative in polymerase chain reaction [17].

Even though serovar Sarawak has not been reported to be present in cattle in Malaysia, there have been several studies reporting the presence of the serovar in wild animals. In a leptospirosis seropositivity prevalence study on wild animals in Sarawak (a state in East Malaysia), it was documented that 72% of the positive samples were also positive for serovar Sarawak [21]. The animals which were positive for serovar Sarawak were bats (*Cynopterus brachyotis, Penthetor lucasi*, and *Hipposideros cervinus*), monkeys (*Macaca nemestrina*, *Hylobates muelleri, Trachypithecus cristatus*, and *Nasalis larvatus*), rats (*Sundamys muelleri*), squirrels (*Callosciurus notatus*), and mongooses (*Herpestes brachyurus*). Rats have been recognized as carriers of *Leptospira* which cause infection in both humans and animals. However, the carrier status of the other wild animals stated above need to be further explored. In our study, wild animals such as squirrels and boars were also sighted in the cattle farms.

Currently, there is no available information regarding the pathogenicity of *L. interrogans* serovar Sarawak (Lepto 175) and its endemicity in Malaysia. There is on-going study conducted by the Institute for Medical Research Kuala Lumpur on the serovar Sarawak [21]. Other local strains such as serovar Melaka and Terengganu also have limited published studies available. Information regarding pathogenicity and the reservoir animals that harbor the bacteria is very limited at the moment. With further research on these local serovars, the source of infection and the route of transmission can be further understood for prevention planning.

Cattle have been reported to be more prone to leptospirosis compared to other animals such as goats. Leptospiral infection patterns in cattle can be separated into two groups based on the infecting serovars. The first group comprises serovars which are carried by and well-fitted to cattle worldwide, such as serovar Hardjo. These serovars are not affected by regional factors or rain patterns. The second group comprises incidental infection by local serovars carried by other animals in the surrounding areas; these serovars are affected by environmental factors and breeding practices. The second group is more commonly found in tropical countries [17].

Farms are mainly located in rural areas with adjacent jungles, which make the presence of wild animals such as boar, bats, squirrel, and others possible. Some of the cattle farmers have stated the presence of these wild animals in their farms. While both livestock and wild animals are known carriers of *Leptospira*, their presence in the farms promotes direct transmission of *Leptospira* by physical contact between animals or indirect transmission through contact with urine. With large numbers and varieties of animals in the farms, it is probable that the transmission of infection can occur through direct contact between the animals.

Among the cattle in this study, the highest seropositivity rate for *Leptospira* antibodies occurred in District A, where all the cattle were positive. The second highest seropositivity rate in cattle occurred in District B, followed by District C, District D, District E, and District F. However, the majority of the cattle were found to be seropositive regardless of the district. Therefore, it can be hypothesized that cattle are carrier hosts for *Leptospira* and have the potential of maintaining serovar Sarawak.

The reason for the seropositivity in all cattle in District A (the eponym of which is also the capital city of Northeastern State of Malaysia) could probably be due to the high urban population density, compared to other districts [22]. A densely populated area attracts more rats [23], which can cause a higher risk for leptospirosis. However, further research is needed before any conclusion can be made regarding this fact.

## Conclusion

The high seroprevalence indicates that cattle are a high-risk animal for leptospirosis. They were exposed to the urine and possibly leptospire-contaminated environment. High seroprevalence among cattle may increase the risk for their owner to also get the infections. *Leptospira* serovar Sarawak is the predominant infecting serovar detected among the seropositive cattle.

## Recommendation

As our study did not include leptospirosis among other animal reservoirs or human, we could not conclude the pattern and interaction between the animal and human. We recommend further studies on human, local animal reservoirs, and wild animals along with the surrounding environment to provide important information on predominant serovar.

## Authors’ Contributions

AD and MMA designed the study. NMHMF, SK, and WMZWM conducted the experimental work, while FA and NI assisted during the laboratory experiment. AD and NMHMF drafted the manuscript and corrected it. All authors read and approved the final manuscript.
